# Selection Bias in Analyses of Physical and Cognitive Function: the Study of Women’s Health Across the Nation

**DOI:** 10.1093/geroni/igaf122.2043

**Published:** 2025-12-31

**Authors:** Lauren MacConnachie, Jillian Baker, Alexis Reeves, Michelle Hood, Kelly Ylitalo, Aleda Leis, Sherri-Ann Burnett-Bowie, Carrie Karvonen-Gutierrez

**Affiliations:** University of Michigan, Ann Arbor, Michigan, United States; University of Michigan School of Public Health, Ann Arbor, Michigan, United States; Stanford Medicine - Stanford University, Stanford, California, United States; University of Michigan School of Public Health, Ann Arbor, Michigan, United States; Baylor University, Waco, Texas, United States; University of Michigan, Ann Arbor, Michigan, United States; Mass General Research Institute, Boston, Massachusetts, United States; University of Michigan, Ann Arbor, Michigan, United States

## Abstract

The bi-directional relationship between physical and cognitive functioning is well-known, but longitudinal investigations may produce biased estimates due to differential loss-to-follow-up caused by poor health or mortality. We hypothesize that selection bias significantly affects estimates of the association between gait speed and cognitive function in the Study of Women’s Health Across the Nation (SWAN). SWAN is a community-based, longitudinal study of 3,302 women. At visit 15, 59.4% (n = 1,345) of participants completed a timed four-meter gait test, East-Boston Memory Test (immediate and delayed), Symbol Digit Modalities Test (SDMT), and Digit Span Backwards test. Linear regressions analyzed the relationship between gait speed and each cognitive test, incorporating inverse probability weighting to correct for selection bias. Models were analyzed with and without weights and stratified by financial strain and education to examine moderation. Weighted models yielded larger effect estimates than unweighted models, especially among disadvantaged women. Among women with a high school education or less, the unweighted model estimated increased gait speed is associated with 17.8 higher SDMT score (β:17.8, 95%CI:11.7, 23.9), while the weighted model estimated the effect as 16.4% larger (β:21.26, 95%CI:15.11, 27.42). Among women with a college degree or more, the weighted effect estimates were only 9.1% larger than unweighted estimates. A similar pattern was observed in models stratified by financial difficulties and for other cognitive outcomes. Selection bias in the SWAN study resulted in underestimation of the association between gait speed and cognitive function, especially among disadvantaged groups. Addressing this bias is essential for accurate estimation of these relationships.

